# Large-scale proteomics reveals precise biomarkers for detection of ovarian cancer in symptomatic women

**DOI:** 10.1038/s41598-024-68249-2

**Published:** 2024-07-27

**Authors:** Emma Ivansson, Julia Hedlund Lindberg, Karin Stålberg, Karin Sundfeldt, Ulf Gyllensten, Stefan Enroth

**Affiliations:** 1 Department of Immunology, Genetics, and Pathology, Biomedical Center, SciLifeLab Uppsala, Uppsala University, 75108 Uppsala, Sweden; 2 Department of Women’s and Children’s Health, Uppsala University, 75185 Uppsala, Sweden; 3 Department of Obstetrics and Gynaecology, Institute of Clinical Sciences, Sahlgrenska Academy at Gothenburg University, 41685 Gothenburg, Sweden

**Keywords:** Ovarian cancer, Diagnostic markers, Proteomic analysis

## Abstract

Ovarian cancer is the 8th most common cancer among women and has a 5-year survival of only 30–50%. While the survival is close to 90% for stage I tumours it is only 20% for stage IV. Current biomarkers are not sensitive nor specific enough, and novel biomarkers are urgently needed. We used the Explore PEA technology for large-scale analysis of 2943 plasma proteins to search for new biomarkers using two independent clinical cohorts. The discovery analysis using the first cohort identified 296 proteins that had significantly different levels in malign tumours as compared to benign and for 269 (91%) of these, the association was replicated in the second cohort. Multivariate modelling, including all proteins independent of their association in the univariate analysis, identified a model for separating benign conditions from malign tumours (stage I–IV) consisting of three proteins; WFDC2, KRT19 and RBFOX3. This model achieved an AUC of 0.92 in the replication cohort and a sensitivity and specificity of 0.93 and 0.77 at a cut-off developed in the discovery cohort. There was no statistical difference of the performance in the replication cohort compared to the discovery cohort. WFDC2 and KRT19 have previously been associated with ovarian cancer but RBFOX3 has not previously been identified as a potential biomarker. Our results demonstrate the ability of using high-throughput precision proteomics for identification of novel plasma protein biomarker for ovarian cancer detection.

## Introduction

Ovarian cancer 5-year survival ranges from 90% when the cancer is discovered in stage I to 20% in stage IV^1^. This partly reflects that more aggressive subtypes tend to be diagnosed at later stages, but it has also been suggested that early detection could improve survival. Ovarian cancer is the 8th most common cancer among women today and kills over 200 thousand women per year worldwide^2^. At present, the discovery of ovarian cancer is symptom-driven, and less than a third of the cases are discovered at stage I or II^1^. Detailed understanding of the aetiology of ovarian cancer could assist in determination of an optimized screening interval in relation to underlying processes, but the precursor states have not yet been precisely identified. For high-grade serous carcinomas for instance, the presumed precursor state STIC (serous tubal intraepithelial carcinomas) can itself develop slowly over decades from the first occurrence of predisposing genetic mutations^3^. Recent molecular evidence obtained from patient material suggests that the transition from STIC to ovarian cancer can occur in a relative short time-span estimated to 6–7 years^4,5^. Computational estimates based on tumour sizes and growth rates^6^ have indicated that ovarian cancer can exist over 4 years in situ, or as stage I and II, before finally progressing to stages III and IV.

Today, no sufficiently accurate enough molecular test exists to justify population-based screening. The largest running prospective ovarian cancer screening study UKCTOCS (United Kingdom Collaborative Trial of Ovarian Cancer Screening)^7^ has evaluated a multi-modal screening strategy for post-menopausal women. This strategy is based on a molecular indication of increased MUCIN-16 (Cancer antigen-125/CA125) which is then followed by a transvaginal ultrasound. Elevated MUCIN-16 was first introduced as an indication for ovarian cancer in 1983^8^ and still remains today the best single value biomarker used both for diagnosis in post-menopause women and for treatment management^9^. A remaining difficulty with the multi-modal approach is the relatively low sensitivity of MUCIN-16 which results in that many cancers are missed. In general, a high specificity can be achieved by analysis of additional biomarkers or by using transvaginal ultrasound (TVU)^10^ which can reduce false positives. Typically, today, women who experience pelvic symptoms are often examined using available molecular biomarker analysis, TVU or computer tomography, and with surgery as the final tool for diagnosis. In Sweden today, close to four out of five women who undergo surgery for adnexal tumours are diagnosed with benign cysts, not cancer^11^ and accurate biomarkers are needed to accurately triage symptomatic women to reduce unnecessary diagnostic surgery. MUCIN-16 as a single biomarker has low sensitivity for early-stage cancer and results in a high rate of false positive indications in many benign gynaecological conditions in younger women, such as infections, pregnancy, or endometriosis^9^. Combinations of MUCIN-16 and additional biomarkers such as the WAP Four-Disulfide Core Domain 2 (WFDC2 or HE4), as used in the ROMA-index (ovarian malignancy risk algorithm) improves the accuracy. The ROMA-index, is calculated differently depending on menopausal status and was initially reported to have a sensitivity of 0.77 at a specificity of 0.75 in pre-menopausal women while a sensitivity of 0.92 at a specificity of 0.75 can be achieved in post-menopausal women^12^. Recent meta-analyses of the ROMA-index in both pre- and post-menopausal women indicates that the overall sensitivity of the test is in the range of 0.88 to 0.93 with a specificity in the range of 0.89 to 0.94^13^. In addition to MUCIN-16 and WFDC2 which are used in the ROMA-index, a number of studies have indicated that additional protein biomarkers can be informative for e.g., triaging or early diagnosis of ovarian cancer. The OVA1-test, combines five proteins (Apolipoprotein A1, Beta 2 microglobulin, CA125, Transferrin and Prealbumin/Transthyretin) and classifies women into categories of high, intermediate or low risk of ovarian cancer and was recently evaluated in a multicentre study^14^ where a higher proportion of the individuals predicted to be low risk, e.g. benign, according to the OVA1-test as compare to using CA125 alone remained benign during a 12-month follow-up period. In a recent study^15^, we showed that combinations of 4 to 7 protein biomarkers selected from over 1450 plasma proteins outperform MUCIN-16 alone in detection of ovarian cancer in symptomatic women. A set of 7 proteins achieved a sensitivity of 0.91 at a specificity of 0.96 separating benign tumours from malign in stage I and II. This high accuracy was then replicated in an independent cohort. Notably, our data-driven approach for selecting biomarkers did not include MUCIN-16 among the 7 proteins showing that a broad characterization of protein biomarkers candidates without prior assumptions on inclusion can break new ground and go far past the current gold standard in molecular tests for early detection of ovarian cancer.

To examine this hypothesis further, we have characterized larger number of plasma proteins in two independent Swedish cohorts consisting of women that underwent diagnostic surgery after suspicion of ovarian cancer and whom were later diagnosed with either benign or malignant tumours.

## Material and methods

### Samples

Plasma samples of women with benign and malignant ovarian tumours were collected from either the U-CAN collection^16^ at Uppsala Biobank, Uppsala University, Sweden or the Gynaecology tumour biobank^17^ at Biobankvast.se, Western healthcare region, Göteborg, Sweden. All samples from the biobanks were included based on surgical ovarian cancer diagnosis or patients that had been surgically diagnosed with benign conditions based on suspicion of ovarian cancer. Exclusion criteria were patients that had received neoadjuvant treatment prior to surgery or if the tumour was pathologically determined to be metastatic originating from other tissues, based on pathology. The samples from U-CAN in Uppsala were collected between 2012 and 2018 in agreement will all local guidelines and regulations. The samples in the Gynaecology tumour biobank in Göteborg were collected from 2016 to 2018 in agreement with all local guidelines and regulations. The tumours were examined by pathologist specialized in gynaecologic cancers for histology, grade, and stage according to International Federation of Gynaecology and Obstetrics (FIGO) standards. Both cohorts contained mixed tumour histology. In the U-CAN samples, among the samples with complete histology data, 60.1% were high grade serous (HGS), 8.7% low grade serous (LGS), 7.6% endometroid, 6.0% clear cell, 5.5% carcinosarcoma and the remainder mucinous, non-epithelial, endometroid or mixed. In the Göteborg samples, 70.6% were HGS, 8.2% LGS, 7.0% mucinous, and the remainder clear cell, endometroid, sarcoma, epithelial/clear cell, mucinous/teratoma or unclear histology. All samples were collected at time of diagnosis, from non-fasting, non-sedated patients by a trained nurse. Separated plasma was then frozen and stored at − 70°C on site. In total, 350 samples were used from the U-CAN collection and 171 from the Göteborg collection. Basic statistics for the samples used are presented in Table 1. The study was approved by the Regional Ethics Committee in Uppsala (Dnr: 2016/145) and Göteborg (Dnr: 201–15) and informed written consent was obtained from all participants following the guidelines of the Declaration of Helsinki.

**Table 1:** Cohort characteristics.

	Cohort	All	Benign	Ovarian cancer			
				I	II	III	IV
Nr. samples	Discovery	171	86	13	11	38	23
	Replication	350	133	46	15	96	60
Age at Diag.^a^	Discovery	60.3 (14.6)	58.1 (16.6)	62.2 (11.5)	67.7 (8.7)	60.3 (12.4)	63.7 (12.4)
	Replication	59.6 (13.4)	56.2 (16.1)	60.9 (11.2)	66.7 (8.4)	61.5 (10.5)	63.2 (11.6)
Age diff pval^b^		0.41	0.19	0.84	0.90	0.93	0.83
Clin. CA-125^c^	Discovery	1174.2 (2382.2)	130.0 (261.1)	133.0 (175.3)	483.4 (648.2)	2032.8 (3133.3)	2389.9 (2995.0)
	Replication	1049.3 (2017.8)	99.3 (157.7)	288.7 (320.3)	n.a	1636.8 (1801.3)	1981.9 (3169.2)
CA-125 diff pval^b^		0.64	0.46	0.48	n.a	0.76	0.31

^a^Reported as mean (std.dev) age in the group. ^b^Two-sided Wilcoxon ranked test comparing the Discovery and Replication cohorts ^c^Clinically measured CA-125 at time of diagnosis, reported as mean (std.dev) U/ml in the group.

### Proteomics

The samples used here have previously been analysed with the proximity extension assay (PEA)^18^ Explore1536^19^ assay^15,20^ and was here studied using the PEA Explore3072 Expansion assay. The samples were randomized across seven 96 well plates. In brief, the PEA is based on pairs of antibodies equipped with DNA single-strand oligonucleotide reporter molecules, probes, that bind to their respective target if present in the sample. Target binding by both probes in a pair in close proximity generates double-stranded DNA amplicons. The Olink Explore3072 Expansion assays is built upon four separate 384-plex panels focusing on Inflammation, Oncology, Cardiometabolic and Neurology proteins, corresponding to a total of 2943 unique human proteins, and the workflow has been described in detail before^21^. After the initial probe-based immune reaction step in the Explore workflow, the amplicons were extended and amplified in a two-step process. Individual sample index sequences were added during the second step. After this step the samples were pooled. Sequencing libraries were prepared and subsequently sequenced on a NovaSeq 6000 instrument (Illumina, USA) according to the manufacturer’s instructions. After sequencing, the generated BCL files were transformed into count files. The count files were then translated into normalized protein expression (NPX) values through a quality control (QC) and normalization process built around internal and external controls as specified by the manufacturer of the assay. The resulting NPX values are on a log2 scale and in the logarithmic phase of the curve, one (1) increase of the NPX value corresponds to a doubling of the protein content. In the resulting data, a high NPX value corresponds to a high protein concentration. Each of the measured proteins has a lower limit of detection (LOD) given in the same NPX-scale which is determined at run time. Here, each protein measure with NPX under LOD was replaced with the plate-specific LOD as indicated in the provided result file. In total, 8.8% of the measurements in the Explore 1536 assay were found to be under the LOD and 31.1% of the measurements in the Explore 3072 expansion asssay. All experimental methods were conducted in accordance with relevant guidelines and regulations.

### Data analysis and statistics

All calculation were carried out using R^22^ (4.2.2). Univariate comparisons were done one protein at a time using a two-sided Wilcoxon ranked based test. The resulting p-values were adjusted for multiple hypothesis testing using the Holm correction method as implemented in the ‘p.adjust’ R-function. For the multivariate analyses, a feature selection was first done using the training cohort only based on recursive feature selection as implemented by the ‘rfe’ function in the ‘caret’ R-package^23^ (version 6.0.91) using ‘nbFuncs’ as functions with method set to ‘repeatCV’ with 4 repeats. The feature selection was carried out by allowing combinations of 2 to 20 individual proteins. A Naïve Bayes model was then trained using the ‘caret’ R-package employing a four-fold cross-validation schema optimising the Laplace correction (‘fL’ parameter) from 0 to 1 in steps of 0.1, with and without kernel and bandwidth adjustment (‘adjust’ parameter) from 1 to 4 in steps of 0.1. The model returned a score in the range 0 to 1 and thresholds (cut-off) for separating the classes was determined by evaluating the receiver operating characteristics (ROC) on the training cohort at a minimum sensitivity and/or specificity of 0.95 and at the ‘best point’ meaning the closest (Euclidian distance) point on the ROC-curve to perfect classification. The model performance was evaluated separately in the training and the validation cohorts. No samples from the validation data were used in the training nor in the optimization in any of the models. The obtained performances in the validation cohort were compared to the obtained performance in the training cohort based on the area under curve (AUC) statistics and the achieved sensitivity and specificity at the cut-off developed in the training cohort. AUC-statistics were compared using the DeLong’s test and a two-sided Fisher’s Exact test on a 2 × 2 matrix with true or false negatives or positives was used to compare the obtained sensitivity and specificity between the training and validation cohort. Correlations between proteins were calculated with Spearmans’s method using the ‘cor.test’-function. Beeswarm-plots were made using the R-package ‘beeswarm’ (0.4.0)^24^. The literature-search was conducted by searching PUBMED (https://pubmed.ncbi.nlm.nih.gov/) for “ovarian cancer” and (i) the protein short-name (ii) the protein full name and (iii) the name of the encoding gene. All statistical analyses were conducted in accordance with relevant guidelines and regulations.

## Results

### Multiple single value biomarkers for early detection

**Figure 1:**
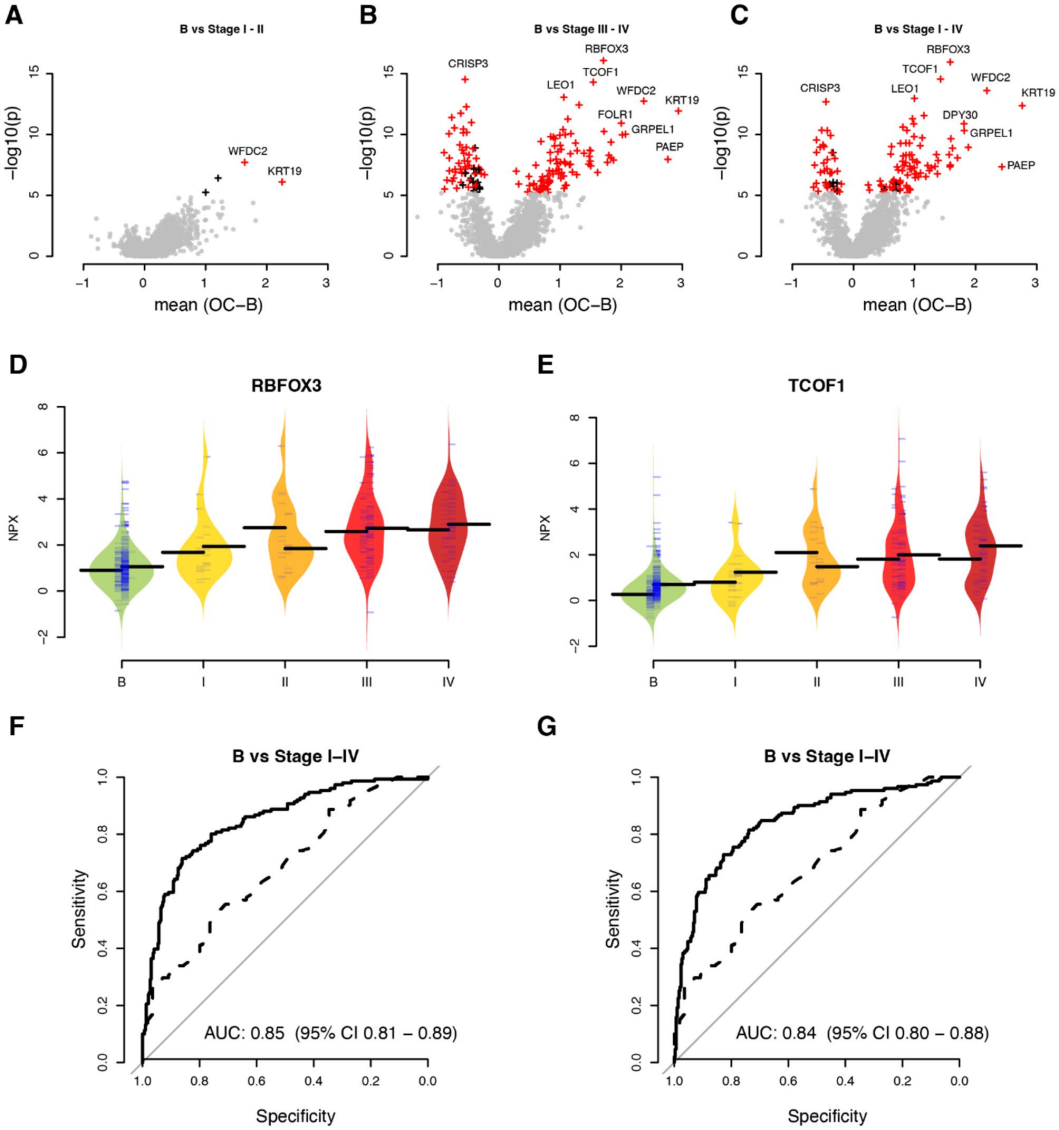
Univariate results (**A**) Analyses (‘volcano plots’) of benign compared to early stage (stage I and II) in the discovery cohort. Mean NPX differences (malign–benign) are shown on the x-axis and p-values (− log10, two-sided Wilcoxon ranked test) on the y-axis. Proteins plotted with a ‘ + ’ were significantly different in the discovery data (q < 0.05, Holm-adjusted). Proteins plotted in red were also found to be significant (q < 0.05, Holm-adjusted) also in the replication data while proteins plotted in black were not. Up to 10 proteins with the lowest p-values in the discovery data and/or the largest NPX-difference are labelled. (**B**) Same as (**A**) but for benign compared to late stage (stage III and IV). (**C**) Same as (**A**) but for benign compared to any stage (stage I–IV). (**D**) Beenplots of individual protein measurements for RBFOX3 in the discovery cohort (left side) and replication cohort (right side). Group mean is indicated with a black line and individual measures with thin blue lines. The samples are divided by diagnose: B—benign (coloured grey), I, II, III and IV—ovarian cancer FIGO stage (coloured yellow to red). (**E**) As (**D**) but for TCOF1. (**F**) ROC-curve for RBFOX3 (solid) and MUCIN-16 (dashed) in the replication cohort using a model developed in the discovery cohort. The AUC is indicated with 95% confidence interval. (**G**) As (**F**) but for TCOF1 and MUCIN-16.

A total of 571 samples from two separate clinical cohorts with women diagnosed with benign or malign tumours based on suspicion of ovarian cancer, was analysed using Explore^19^ PEA^18^. The first set of samples (Table 1, discovery cohort) were from a cohort collected in Göteborg, Sweden and the second (Table 1, replication cohort) from the U-CAN biobank in Uppsala, Sweden). Here, 443 samples were analysed using the Olink Explore3072 expansion assay. Part of the samples have previously been characterized with the Olink Explore1536 assay and results from these analyses have been published^15,20^. Both panels (1536 and 3072) each contains 1536 assays. One-hundred and seven (107) samples from the discovery cohort and 163 samples from the replication cohort had complete data from both analyses runs. In our previous analysis^15^ the Göteborg cohort was used as discovery cohort while the Uppsala cohort was used for replication and the same assignment was used here. We first analyzed the data from the two PEA Explore assays separately to maximize the statistical power in the univariate analysis. This included up to 111 and 237 samples from the discovery and replication cohorts respectively for the Explore1536 assay, and 167 and 276 samples from the discovery and replication cohorts for the Explore3072 assay. The univariate analyses were performed in three ways; benign vs early-stage ovarian cancer (stages I and II), benign vs late stage (stages III and IV) and finally benign vs any stage (I-IV). After adjustment for multiple-hypothesis testing in the discovery cohort, a total of 4 proteins were found to be significantly different between benign and early-stage cancer (Fig. 1A), 163 between benign and late-stage (Fig. 1B) and finally, 129 between benign and any stage (Fig. 1C). Several of these 296 proteins differed significantly in more than one category of comparisons, and in total 171 unique proteins were involved. We then analysed the 296 associations in the replication cohort and found that all 296 associations had fold-changes in the same direction also in the replication cohort and that 279 (94.3%) of these were nominally significantly different. After adjustment for multiple-hypothesis testing also in the replication cohort, 269 (90.9%) of the associations remained significant. All univariate results are presented in Supplementary Table 1. Here, MUCIN-16 was found to be among the biomarkers with replicated performance, but ranked as the 139th most significant association by p-value in the discovery data when comparing benign and any stage cancer. RBFOX3 and TCOF1 were the two proteins with the lowest p-values comparing benign with any stage cancer and the distribution of obtained NPX values in both the discovery and replication cohorts for these by stage are shown in Fig. 1D and E. Next, we compared the performance of these two biomarkers as single valued classifiers for separating benign from any stage. In the replication cohort, RBFOX3 had an AUC (area under curve) of 0.85 (95% confidence interval 0.81–0.89, Fig. 1F) and TCOF1 had an AUC of 0.84 (0.80–0.88, Fig. 1G) while an AUC of 0.68 (0.61–0.75) was found for MUCIN-16 as measured by the PEA. In Figures F and G, the selected biomarker is shown as a solid line while the performance of MUCIN-16 (as measured with the PEA) is shown as a dashed line. For both proteins, the AUC was significantly higher than for MUCIN-16 (all p-values < 1.1 × 10^–4^, DeLong’s test). Using both cohorts, the clinical CA125 measurements were found to have a moderate (Spearman’s’ Rho = 0.53) but significant (p < 2.8 × 10^–9^) correlation with the PEA equivalent. Across both cohorts, the CA125 measurements achieved an AUC of 0.70 (0.56—0.85), 0.93 (0.87–0.98) and 0.87 (0.80—0.94) in separating benign from early stage (I and II), late stage (III and IV) and any stage (I-IV) tumours, respectively. At the often clinically used cutoff at 35 U/ml, CA125 alone achieved sensitivities of 0.78 (0.61–0.94), 1.00 (1.00–1.00) and 0.94 (0.89–0.99) and specificities of 0.44 (0.31–0.59), 0.44 (0.28–0.59) and 0.44 (0.28–0.59) respectively for the same three categories.

### Combining biomarkers increases precision

We next built multivariate prediction models separating; (i) benign vs early stages (I and II), (ii) benign vs late stages (III and IV) and (iii) benign vs any stage (I-V). For all three models, we used the same methodology where we first employed feature selection based on all proteins in the discovery cohort. The selected proteins were then used to build and optimize a classifier reporting a risk-score on the scale of 0 to 1. We then set a cut-off for predicting malignancy based on the risk-score where we aimed to achieve at least 95% sensitivity. We then applied the model to the replication cohort calculating the risk-score applying the same cut-off and evaluated the final performance of the models based on those scores only. In this analysis, the data from the two PEA assays (Explore 1536 and the 3072 expansion, see Methods for details, was merged keeping only proteins and individuals with no missing values. This resulted in a final data set with 2934 proteins measured in both cohorts, 107 samples in the discovery cohort and 163 samples in the replication cohort.

**Figure 2:**
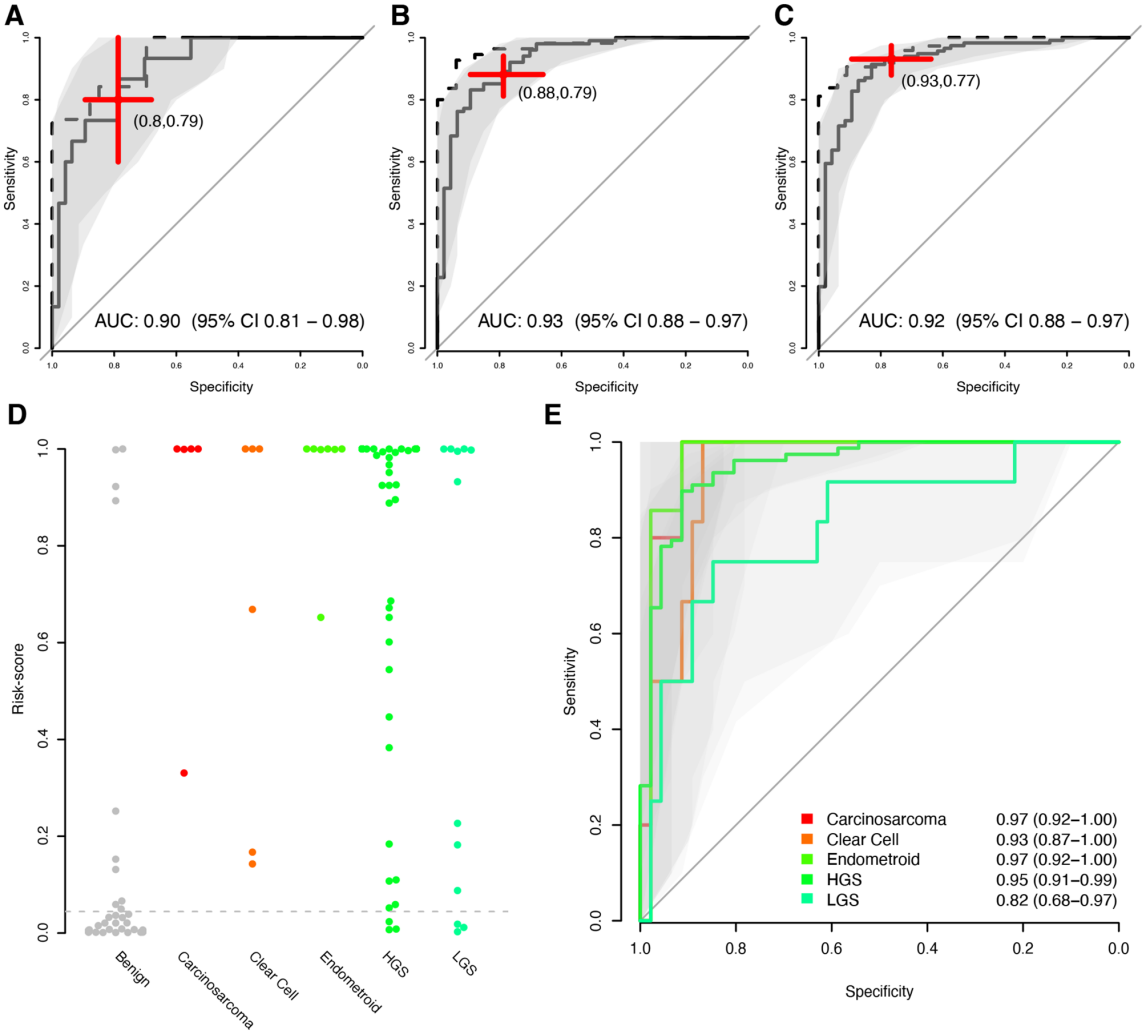
Multivariate performance in the replication cohort. (**A**) ROC curves for the performance in the replication data (solid black line) and in the discovery cohort (dashed curve) separating early stages (I and II) from benign. The shaded area corresponds to 95% confidence interval for the ROC curve in the replication cohort. The red cross is centred on the point estimate of the sensitivity and specificity in the replication cohort for the cut-off obtained at least 95% sensitivity in the discovery cohort. The height and width of the crosses illustrate the 95% confidence intervals of sensitivity (y-axis) and specificity (x-axis). (**B**) As (**A**) but for late stages (III and IV) vs benign. (**C**) As (**A**) but for any stage (I–IV) vs benign. (**D**) Distribution of risk-scores in the replication cohort as calculated by the model in (**C**) for specific tumour histologies and benign. The cut-off for 95% sensitivity is indicated by a vertical dashed grey line. (**E**) As (**C**) but separated based on tumour histology (same categories as in (**D**)). Only ROC-curves for the replication cohort are shown. AUCs are written out as estimate and 95% confidence intervals.

The model separating early stages from benign consisted of three proteins, WFDC2, FOLR1 and KRT19, and achieved an AUC of 0.90 in the replication cohort (Fig. 2A) which was not statistically different from the performance in the discovery cohort (AUC = 0.94, p = 0.45, DeLong’s method, Table 2), suggesting robust modelling performance. Compared to the performance of CA125 alone for separating early-stage cancers from benign, the three-protein model achieved significantly (p < 0.023, DeLong’s method) higher AUC. The feature selection for the models for late stages vs benign were built using two proteins, RBFOX3 and WDFC2, and achieved and AUC of 0.93 in the replication cohort (Fig. 2B), as compared to 0.97 in the discovery cohort (p > 0.12, DeLong’s method, Table 2). Finally, the model for separating benign from any stage consisted of three proteins, WFDC2, FOLR1 and KRT19, and had an AUC of 0.92 in the replication cohort (Fig. 2C), as compared to 0.97 in the discovery cohort (p > 0.12, DeLong’s method, Table 2). Although the AUCs achieved by the multivariate protein models comparing late (III and IV) and any stage (I-IV) vs benign were consistently higher than for CA125 alone, these differences were not statistically significant (p > 0.19, DeLong’s method). Lastly, using the discovery cohort we also developed a cut-off for each model requiring at least 95% sensitivity in separating the malign from the benign which was then applied also to the replication cohort. For all three models, there was no statistical difference (all p-values > 0.08, Fisher’s Exact test, Table 2) in the point-estimates of sensitivities and specificities obtained in the replication data at the respective cut-off. Overall, the three models generated here for early, late and any stage vs benign conditions contained only four proteins in total (WFDC2, FOLR1, KRT19 and RBFOX3). We also compared the performance of the benign vs any stage model for separate tumour histologies (Fig. 2D). When comparing the raw risk-scores between histologies, we found nominally significant (two-sided Wilcoxon ranked based test) differences between Carcinosarcoma (p = 0.037), Endometroid (p = 0.022) and HGS and LGS (p = 0.037, 0.022 and 0.0021 respectively) with lower values in the LGS (Fig. 2D). After adjustment for multiple hypothesis testing, only the difference between HGS and LGS remained significant (q = 0.021). In addition to the raw risk-score distribution, we also compared the fraction of samples predicted as false negatives in the different histologies and found a nominally significant (p = 0.029, Fisher’s exact test) higher proportion in LGS as compared to HGS although this significance did not remain after adjustment for multiple hypothesis testing (q = 0.29). Lastly, we compared the predictive performance of the model in separating benign from specific histologies (Fig. 2E) and although there is a trend of worse performance in separating LGS from benign as compared to all other comparisons (Fig. 2E), we found no statistical difference in the estimated AUC between any of the categories (all nominal p-values > 0.063, DeLong’s method).

**Table 2:** Results of multivariate modelling.

		AUC^a^	Sens^b^	Spec^b^
B vs I–II	Discovery	0.94 (0.88–1.00)	1.00 (1.00–1.00)	0.70 (0.55–0.85)
	Replication	0.90 (0.81–0.98)	0.80 (0.60–1.00)	0.79 (0.68–0.89)
	p-value^c^	0.45	0.08	0.43
B vs III–IV	Discovery	0.97 (0.94–1.00)	0.96 (0.91–1.00)	0.85 (0.73–0.97)
	Replication	0.93 (0.88–0.97)	0.88 (0.81–0.94)	0.79 (0.66–0.89)
	p-value^c^	0.12	0.14	0.57
B vs I–IV	Discovery	0.97 (0.94–0.99)	0.96 (0.91–1.00)	0.73 (0.58–0.88)
	Replication	0.92 (0.88–0.97)	0.93 (0.88–0.97)	0.77 (0.64–0.89)
	p-value^c^	0.12	0.53	0.79

^a^Numbers for discovery and replication are given as a point estimate and 95% confidence intervals. ^b^The point estimate and 95% confidence interval is given at a cut-off defined in the discovery cohort at the point on the ROC (receiver operating characteristics) curve closest to perfect classification. ^c^p-values for difference in AUCs were calculated using the DeLong’s method. For differences of sensitivity and specificity, a Fisher’s exact test was used. B—Benign. Roman numerals specify ovarian cancer stage.

## Discussion

There is a strong need to identify biomarkers for ovarian cancer, both to improve the diagnosis of women seeking healthcare and for population screening. A biomarker test separating benign from malign tumours in symptomatic women could reduce the need for diagnostic surgery and thereby reduce the risk of side effects on fertility and iatrogen menopause. Population screening could enable early detection of women at risk and provide an opportunity to identify early-stage cancer with a better prognosis. Today, none of the available biomarkers is accurate enough in a screening scenario to safely identify close to all cancers (high sensitivity) without also including a considerable fraction of false positives (low specificity). False positives lead to unnecessary anxiety among women until a benign or healthy diagnosis have been confirmed. In ovarian cancer, the diagnostic work-up results in additional examination and diagnostic surgery, which introduce health care costs and may result in complications and anxiety for the women. The largest running ovarian cancer screening study, the UKCTOCS (United Kingdom Collaborative Trial of Ovarian Cancer Screening)^7^, has suggested using multi-modal testing based on elevated MUCIN-16 followed by transvaginal ultrasound in post-menopausal women. Health-economic studies support that this strategy could be justified for screening^25^. Recently, the long-term outcome in the UKCTOCS study^26^ was analysed, and although an increase in early-stage cancer discovery was observed, no clear improvement could be seen in reduced mortality. A similar study in the United States, the Normal Risk Ovarian Screening Study (NROSS), which employed the same multi-model strategy did, however, find more promising results^10^. A test based on multiple biomarkers could potentially achieve both a high sensitivity and specificity and thereby complement single CA125 or even replace the multi-modal strategy if the accuracy of a test is high enough. For symptomatic women in Sweden today, where a TVU indicates adnexal ovarian mass, surgery is used for final diagnosis but close to 80% of these women have benign conditions. In this scenario, a molecular test with a high sensitivity and a moderate specificity would still be useful for triaging patients and could therefore reduce unnecessary surgery. The OVA1-test achieves a high sensitivity (above 90%)^27,28^ at a specificity of 49–69%^27,28^ and has in a multicentre study been shown to be clinical useful when compared to using CA125 alone^14^. Here, we show that a biomarker signature based on 3 proteins can separate malign from benign conditions at a sensitivity of 93% while retaining a specificity of 77%. This biomarker panel could potentially reduce the number of women in need for diagnostic surgery with up to two thirds, saving health care resources and reducing the risk of complications for the women.

Our investigation is based on close to 3000 characterized proteins using a machine learning based approach to select proteins to be included in the prediction models, without prior assumption regarding known association with ovarian cancer, nor restriction to proteins with high univariate significance. We have previously developed^29^ and validated^30^ a plasma protein biomarker panel consisting of 11 proteins for detection of ovarian cancer. That panel was selected from analyses of up to 983 proteins and comparing benign and malign tumours achieved an AUC of 0.92 and 0.93 in the validation stage of two separate cohorts comparing benign and malign tumours. In the present study, three out of the four proteins in the final models were also part of the previous 11-biomarker panel with the fourth being exclusively present on Explore3072-expansion and not available in our previous analyses. Although these three proteins were measured using the same technology (PEA), it should be noted in our studies, they were characterized using two different versions of the PEA; the Target 96 and the Explore. Target 96 has a PCR-based readout while the Explore assays uses next-generation sequencing. Although similar, the manufacturers webpage lists slightly different performances for the two techniques, with somewhat lower %CVs for the Explore versions but also slight differences in the expected measuring ranges. This in turn could affect the performance of the individual proteins assays as part of a combined biomarker panel. On the other hand, the fact that the same three proteins were selected as top candidates, independent of the version of the PEA technology used, testified to the robustness of the technology as well as strength of the association with ovarian cancer.

The models presented here were exclusively generated using a discovery cohort followed by validation in an independent replication cohort. The detected models showed similar performance in the two cohorts, suggesting robust behaviour without overestimation of the predictive capabilities. Only four proteins were selected to be included in the final models for separating early, late and any stage from benign conditions.

In a clinical setting, the need to decisively distinguish between only early or late stage from benign conditions is likely not common why the any-stage model is likely to be the most relevant. Notably, neither model developed here included MUCIN-16 even though the protein was available for all models in the feature selection step. As reported above, when comparing the clinically reported CA125 values to the MUCIN-16 as measured by the PEA, we found a moderate but significant correlation between the values of two assays. Technical differences in analytical range between the assays could be a factor in why MUCIN-16 was not included in the models generated. Apart from technical factors, it should also be noted that the comparisons made was between malign and benign conditions and that MUCIN-16 is known to be elevated in several benign gynaecological conditions^9^. This is clear from the clinically measured levels (Table 1), where the mean values among patients with benign diagnoses in both cohorts (130.0 and 99.3 U/ml) are well above the established cut-off at 35 U/ml for ovarian cancer. Several of the four proteins used in our multivariate models here have previously been proposed as biomarkers for ovarian cancer. WFDC2 (also known as HE4) is part of the ROMA-score, and high expression of both KRT19 and FOLR1 has been associated with poor outcome and progression in ovarian cancer^31,32^. RBFOX3 (RNA binding fox-1 homolog 3) has a known function in the regulation of alternative splicing of pre-mRNA, is commonly expressed in the central nervous system^33^, and has been implicated in for instance neuroblastoma^34^ and reported as elevated in prostate cancer ^33^. The role of RBFOX3 in ovarian cancer is, however, not well understood and we have not found any previous relating literature linking to ovarian cancer.

We analysed a large set of proteins with a commercial assay in two independent cohorts from two different geographical locations. A major strength of our study is the strict use of one cohort as discovery and the second for replication, both for the univariate and multivariate analyses. These cohorts contained a mixture of histology diagnoses both among the malignant and benign samples which is reflective of the distribution that should be targeted in a future screening scenario. Our study is however limited by the sample size from the cohorts and the distribution of analysed proteins, reducing the samples available for complete analyses. The sample size also prohibited us from performing detailed stratified analyses of different histologies and/or cancer stages, which could have further improved our models. Our results are also limited by that we are only analysing Swedish samples and that the study does not include neither symptom-free controls nor samples collected before diagnosis, that could have been used to investigate the performance of the developed risk-score in a screening scenario.

Recent advances in the throughput of ultra-highly sensitive proteomics technologies such as the one used here, makes it possible to characterize an increasingly higher number of plasma proteins using very small quantities of input material. Coupling such analysis technologies with machine learning approaches to detect combinations of biomarkers with robust predictive power is a powerful approach to break new ground and go beyond the current knowledge. The PEA technology has been shown to work well not only in wet plasma but also from dried blood spots^35,36^. This opens the possibility of screening based on self-collected dried blood spots, coupled with precise molecular biomarkers as a cost-efficient solution for early detection and monitoring of ovarian cancer.

### Supplementary Information

Supplementary Table 1.

## Data Availability

Raw data is located in controlled access data storage at the Swedish Science for Life Laboratories (SciLifeLab) Data Repositories accessible at https://doi.org/10.17044/scilifelab.25237765.
